# Low-Volume *Ex Situ* Lung Perfusion System for Single Lung Application in a Small Animal Model Enables Optimal Compliance With “*Reduction*” in 3R Principles of Animal Research

**DOI:** 10.3389/ti.2024.13189

**Published:** 2024-09-09

**Authors:** K. Katsirntaki, S. Hagner, C. Werlein, P. Braubach, D. Jonigk, D. Adam, H. Hidaji, C. Kühn, C. S. Falk, A. Ruhparwar, B. Wiegmann

**Affiliations:** ^1^ Department for Cardiothoracic, Transplantation and Vascular Surgery, Hannover Medical School, Hannover, Germany; ^2^ Lower Saxony Center for Biomedical Engineering, Implant Research and Development (NIFE), Hannover, Germany; ^3^ Institute of Pathology, Hannover Medical School, Hannover, Germany; ^4^ Member of the German Center for Lung Research (DZL), Biomedical Research in Endstage and Obstructive Lung Disease Hannover (BREATH), Hannover, Germany; ^5^ Institute of Pathology, RWTH Aachen Medical University, Aachen, Germany; ^6^ Institute of Transplant Immunology, Hannover Medical School, Hannover, Germany

**Keywords:** rat model, *ex vivo* lung perfusion/EVLP, *ex situ* lung perfusion/ESLP, small animal model, single lung

## Abstract

*Ex situ* lung perfusion (ESLP) is used for organ reconditioning, repair, and re-evaluation prior to transplantation. Since valid preclinical animal models are required for translationally relevant studies, we developed a 17 mL low-volume ESLP for double- and single-lung application that enables cost-effective optimal compliance “reduction” of the 3R principles of animal research. In single-lung mode, ten Fischer344 and Lewis rat lungs were subjected to ESLP and static cold storage using STEEN or PerfadexPlus. Key perfusion parameters, thermal lung imaging, blood gas analysis (BGA), colloid oncotic pressure (COP), lung weight gain, histological work-up, and cytokine analysis were performed. Significant differences between perfusion solutions but not between the rat strains were detected. Most relevant perfusion parameters confirmed valid ESLP with homogeneous lung perfusion, evidenced by uniform lung surface temperature. BGA showed temperature-dependent metabolic activities with differences depending on perfusion solution composition. COP is not decisive for pulmonary oedema and associated weight gain, but possibly rather observed chemokine profile and dextran sensitivity of rats. Histological examination confirmed intact lung architecture without infarcts or hemorrhages due to optimal organ procurement and single-lung application protocol using our in-house-designed ESLP system.

## Graphical Abstract

**Figure:**
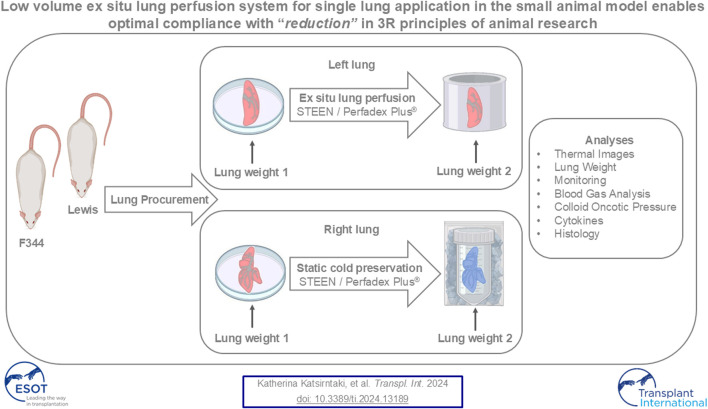
After lung procurement from Lewis and Fischer 344 rats, the left lung was perfused *ex situ*, while the right lung was kept statically cold as a control. In addition to thermal imaging, lung histology and analyses of lung weight, key perfusion parameters, blood gas analysis, cytokines, and colloidal oncotic pressure were performed. Figure was created with BioRender.com.

## Introduction

For patients with end-stage lung disease, lung transplantation (DLTx) currently remains the only curative treatment option and is reserved for highly selected patients, particularly due to the increasing mismatch of potential organ donors to recipients [1, 2]. However, DLTx is also associated with many challenges, specifically challenges surrounding the gold standard of static cold preservation (SCS), which contributes to limited ischemic time and ischemia/reperfusion injury (IRI), which in turn result in acute life-threatening graft failure. This is what leads to the sobering 5-year survival rate of approximately 60% [2]. To address these problems, there is a global focus on *ex situ* lung perfusion (ESLP), which allows temperature-adapted perfusion with blood-like preservation solutions in an almost physiological milieu [3–6]. This ensures *ex situ* the maintenance of organ function and, compared to SCS, shows significantly better outcomes after DLTx [3–6]. This is based in particular on the significant reduction of ischemia time, which in turn decisively minimizes IRI and allows longer organ preservation and thus greater geographic flexibility for donor pool expansion. In addition, diagnosis-dependent *ex situ* therapies and consecutive re-evaluations can be used to convert non-transplantable organs into transplantable organs [7, 8]. The two most common ESLP systems in clinical use are the portable Organ Care System Lung (TransMedics, Inc.; OCS), which uses PerfadexPlus solution with an open left atrium (LA) strategy and allows direct implantation of the harvested lung in the donor hospital, and the XPS system (XVIVO Perfusion; XPS) [4, 5, 8], which uses STEEN™ solution with a cannulated LA as a closed circuit according to the Lund or Toronto protocol, whereby the explanted lung is initially stored statically hypothermically, which is the gold standard of organ preservation and bridges the time of transport from the donor hospital to the recipient hospital where the XPS is stationed [9]. In addition, the three protocols differ in terms of the time at which ventilation is started, the tidal volume and respiratory rate applied, and the perfusion rate and mean pulmonary artery pressure (mPAP) value to be tolerated [4, 5, 8, 9].

In the last decade, there has been a significant increase in the number of studies using these ESLP techniques, some aiming to establish its application for autologous and xenogeneic application, for example, for tumor and infection therapies, as well as for surgical, regenerative, and immunomodulatory procedures [8, 10]. This is accompanied by efforts to create preclinical animal models of ESLP for significant translational studies [11, 12]. These include both large and small animal models, especially rat models, which are characterized by relevant advantages over porcine models [12, 13]. Most researchers working with these rat models use the commercially available Harvard apparatus [12–16], which is technically similar to the non-portable, closed XPS system and is used to mimic the Lund or Toronto protocol, whereby the double lung is subjected to cold ischemia prior to ESLP [12, 14, 15].

Summarized, this illustrates the gap in ESLP systems for rats, because to our knowledge there is currently no corresponding system that can adequately simulate the OCS technique, nor are there corresponding baseline data that initially compare the gold standards around ESLP. These data are essential to enable a detailed and reliable differentiation between ESLP- and SCS-induced as well as *ex situ* perfusion- and transplantation-associated changes in subsequent experiments [4, 5, 17–19]. In line with these requirements, we have established our ESLP, in which we have placed great emphasis on the development of a comparatively cost-effective system during technical optimization that enables optimal compliance with the increasingly relevant reduction of the 3R principles (replacement, reduction, refinement) of animal research.

## Materials and Methods

### Rats, Husbandry and Care

All animal experiments were performed in strict accordance with the German Animal Welfare law and approved by the local authority. For the following experiments, adult male Lewis (Lew) (weight 269 ± 14g) and Fischer344 (F344) (weight 236 ± 27g) rats were housed in ventilated cage systems at 22°C ± 2°C and 55% ± 10% humidity with a 12 h dark/light cycle and were allowed free access to rat chow feed and water *ad libitum*.

### Lung Procurement

Rats were anesthetized with 5-Vol% isoflurane-oxygen mixture, followed by subcutaneous analgesia, consisting of Butorphanol 2 mg/kg/body weight (BW) and Meloxicam 1 mg/kg/BW. Thereafter, orotracheal intubation was performed using a 14-gauge intravenous cannula so that mechanical, volume-controlled ventilation could be initiated with the following ventilatory settings: inspired oxygen fraction of 100%, respiratory rate (RR) 100/min, tidal volume (TV) 6 mL/kg/BW, inspiration:expiration (I:E) time of 1:2, and positive end-expiratory pressure (PEEP) 5 cmH_2_O. After transferring the rat to the temperature-controlled heating mat, intraperitoneal anesthesia was performed with ketamine 60 mg/kg/BW and medetomidine 125 μg/kg/BW, so isoflurane-oxygen mixture was reduced to 1-Vol%. Median laparotomy was conducted to expose the abdominal aorta and inferior vena cava; 1,000IU/kg/BW heparin sodium was administered. Baseline arterial and venous blood was collected, specifically for blood gas analysis (BGA), cytokine analysis, and colloid oncotic pressure (COP) determination, followed by exsanguination. Median sternotomy was performed and thermal baseline lung imaging was taken. In preparation for pulmonary flush, the ascending aorta and pulmonary trunk were separated so that a suture could be placed around the pulmonary trunk to secure the 16-gauge cannula, which was inserted into the right outflow tract via the right ventricle. After cutting superior and inferior vena cava and opening the LA, lungs were flushed pump-controlled with weight-adjusted 4°C cold PerfadexPlus (100 mL/kg/BW), containing 0.25 μg/kg/BW Ventavis and 12 μL mL Tris(hydroxymethyl)aminomethane (THAM). After the second thermal lung imaging, the trachea was clamped and heart-lung block removed and placed on a Petri dish to separate the left and right lungs. Following weight determination and photo documentation, the right lung was stored in 4°C cold PerfadexPlus or STEEN, while the left lung was used for ESLP for the same period of 120 min (min). For this, a 24-gauge cannula was fixed in the pulmonary artery and a 16-gauge cannula was inserted via the left main bronchus to adapt the lung to ESLP.

### *Ex Situ* Lung Perfusion

#### In-House-Designed ESLP System

**FIGURE 1:**
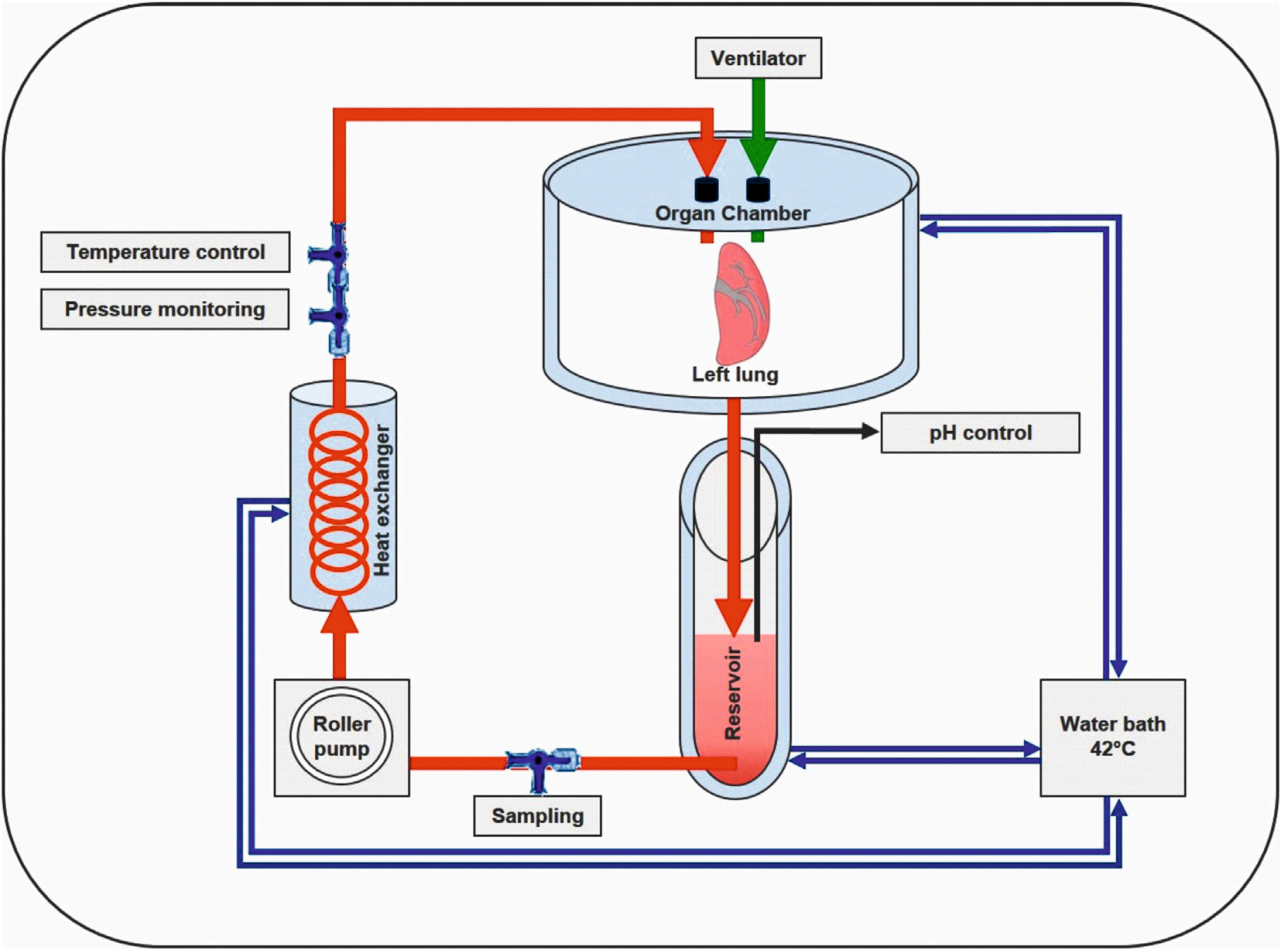
Schematic overview of the in-house-designed rat ESLP system. The left lung of Lewis and Fischer 344 rats was treated with ESLP for 120 min, while the right lung was treated with SCS for the same period of time. The circuit comprised the double-walled organ chamber, the reservoir with the corresponding pH control unit, the roller pump, the heat exchanger, and the water bath. Temperature and pressure were controlled via integrated three-way stopcocks, which were connected to the corresponding monitoring system.

A schematic overview of our in-house-designed ESLP for single- or double-lung application is depicted in Figure 1. The system consists of the following components, whose functions are harmonized and can be monitored separately. Briefly described, the retrieved left lung was connected to the lid of the organ chamber via arterial and bronchial cannulas for single-lung perfusion. Starting from the 42°C thermostatic water bath, heated water circulated through the double-walled organ chamber, the reservoir, and the in-house-designed heat exchanger to keep both the perfusate and lungs at a constant temperature. Perfusion rate was set via the built-in pump and ventilation via the integrated respirator. By integrating three-way stopcocks, both blood sampling and monitoring were possible and supplemented by a digital pH meter in the reservoir and an integrated digital thermometer. Components were connected by silicone tubes, resulting in a priming volume of 17 mL.

#### ESLP

The circuit was primed with PerfadexPlus or STEEN, and samples were taken to determine baseline perfusate values. Antegrade perfusion was initiated at a flow rate of 0.675 mL, which was gradually increased to 3–4 mL/min while maintaining systolic pulmonary artery pressure (PAP) below 30 mmHg. After 5 min ESLP, the first samples were taken and documented every half or full hour, except cytokine analysis, which was only performed at the beginning and end of ESLP. After 15 min, thermal lung imaging was taken before starting ventilation at half TV using the gas mixture of O_2_ (12%), CO_2_ (5.5%), and N_2_ (82.5%), corresponding to the preservation gas during OCS procedure [4–6]. pH was kept between 7.35 and 7.45 by continuous monitoring and adjusted addition of THAM.

In parallel, the right lung was placed under SCS and preservation solution was taken at different time points for BGA, COP, and cytokine analyses. After 120 min, the experiments were terminated, and the left and right lungs were photographed and weighed before being stored in 4% buffered formalin for histomorphological analysis.

#### Analyses

*Thermal lung imaging* was taken at the time points indicated using the Infra Tec VarioCam HDx research 645S infrared camera, offering a geometric resolution of 79,620 pixels per image. To calculate average lung surface temperature, thermal data at each lung pixel point was recorded and analyzed using IRBIS 3.1 plus software before tracing the lung structure under investigation to extract and calculate the corresponding thermal data.

*Lung weight* was determined on a scale at the beginning and end of each ESLP and SCS procedure. Weight gain was calculated as: weight_beginning_ – weight_end_.

*Monitoring analyses* associated with ESLP included the pump-generated perfusate flow, PAP using the connected pressure transducer, pH, and perfusate temperature. The perfusion solution was adjusted to a constant pH of 7.35–7.45 using THAM.

*Perfusate analyses* comprised BGAs, including analyses of partial pressures of oxygen (pO_2_) and carbon dioxide (pCO_2_), as well as potassium, sodium, glucose, and lactate using the ABL90 FLEX blood gas analyzer. The colloidal oncotic pressures were measured with the BMT 923 oncometer.

*Various cytokines* were collected in the perfusate and centrifuged at 1,800 rpm for 5 min at 4°C. Supernatants were then stored at −20°C until they were analyzed using the Bio-Plex Pro Rat Cytokine Group I Panel.

For *morphohistological assessment*, lungs were embedded in paraffin, and 2 µm thick sections were made and stained with hematoxylin and eosin (HE). Semi-quantitative assessments of atelectasis, oedema (perivascular, intra-alveolar, and interstitial), dilated alveolar spaces, and signs of intravascular perfusion solution were performed, with the experimental setup and rat strain blinded and all findings scored 0 (absent) and 1 (present).

### Statistical Analyses

Statistical analysis was performed using GraphPad Prism version 9 (San Diego, United States). Data sets from five independent replicates (n = 5) were used for each analysis. All numerical data were expressed as mean and standard deviation (SD) or standard error of the mean (SEM) and analyzed for significance using two-way ANOVA with Tukey multiple comparison test or one-way ANOVA with non-parametric Kruskal-Wallis and Dunn multiple comparison test for weight gain. *P* < 0.05 was considered statistically significant and marked with corresponding asterisks (*p < 0.05, **p < 0.01, ***p < 0.001, ****p < 0.0001).

## Results

### Key Perfusion Parameters Confirm Valid ESLP for Single-Lung Application

**FIGURE 2:**
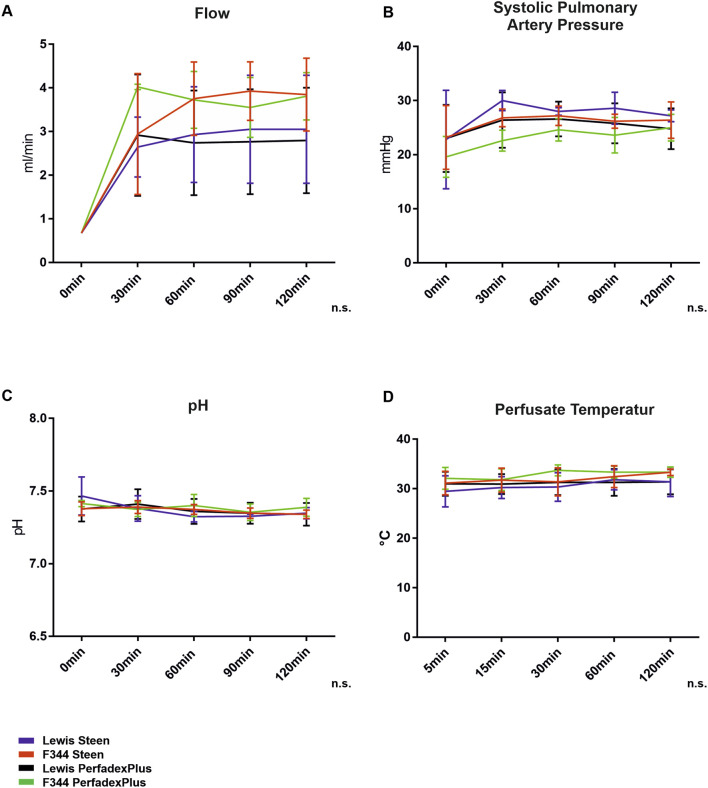
ESLP monitoring. Applied pump flows **(A)**, systolic pulmonary arterial pressures **(B)**, pH **(C)**, and perfusate temperatures **(D)** were recorded in the left lung during ESLP. Data are expressed as mean ± SD. Statistical significance is indicated if *p* < 0.05 or as “n.s” if there is no significance.

All 20 single lungs showed stable key perfusion parameters over the entire ESLP period without significant differences (Figure 2). Perfusate flow was successively increased to the target flow within 30 min and remained constant throughout ESLP (Lew/STEEN 2.471 ± 1.018 mL/min; F344/STEEN 3.0 ± 1.4 mL/min; Lew/PerfadexPlus 2.4 ± 1.0 mL/min; F344/PerfadexPlus 3.2 ± 1.4 mL/min; Figure 2A). Systolic PAP increased in parallel and reached its maximum perfusate flow and remained constant (Lew/STEEN 27.3 ± 2.7 mmHg; F344/STEEN 26.0 ± 1.6 mmHg; Lew/PerfadexPlus 25.3 ± 1.5 mmHg; F344/PerfadexPlus 23.1 ± 2.2 mmHg; Figure 2B). THAM addition secured physiological pH at all times (Figure 2C), whereas test groups with STEEN required significantly less THAM than PerfadexPlus groups (Lew/STEEN 18.0 ± 11.2 µL; F344/STEEN 10.0 ± 11.0 µL; Lew/PerfadexPlus 57.0 ± 25.0 µL; F344/PerfadexPlus 53.4 ± 12.8 µL). Mean perfusate temperature remained at stable levels of 30.7°C ± 0.9°C for Lew/STEEN, 32.0°C ± 0.9°C for F344/STEEN, 31.2°C ± 0.2°C for Lew/PerfadexPlus, and 32.9°C ± 0.8°C for F344/PerfadexPlus (Figure 2D).

### Optimal Thermoregulation Ensures Uniform Lung Temperature as a Sign of Homogeneous Lung Perfusion

**FIGURE 3:**
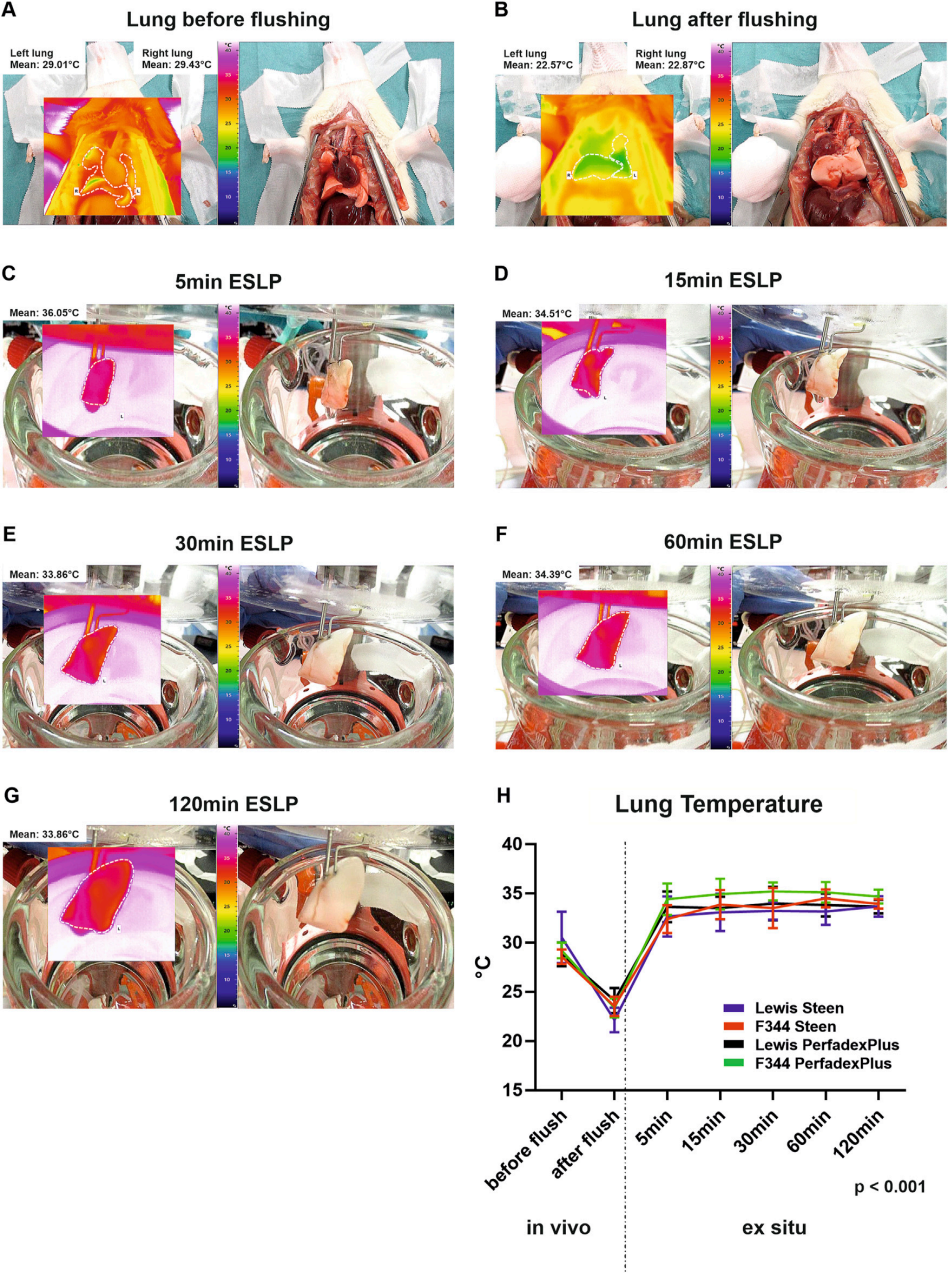
Thermographical data acquisition and analysis of the *ex situ* perfused lungs. Representative native photos and corresponding thermal images of the lungs are shown at various time points as well as the respective average temperature of the lung surface **(A–G)**. Graphical representation shows the surface temperatures of the different test groups over time **(H)**. Data are expressed as mean ± SD. Statistical significance is indicated if *p* < 0.05 or as “n.s” if there is no significance.

Representative thermal lung imaging is shown in Figures 3A–G, indicating a first temperature drop down to approximately 22°C due to cold flush (Figures 3A, B). Left lungs show stable surface temperatures as early as 5min after ESLP start, which averaged 29.01°C (Lew/STEEN 31.2°C ± 4.1°C; F344/STEEN 31.5°C ± 4.0°C; Lew/PerfadexPlus 30.5°C ± 3.1°C; F344/PerfadexPlus 32.5°C ± 4.5°C; Figure 3C) and remained constant over the entire ESLP duration (Figure 3H). There was only one significant difference between F344/PerfadexPlus (32.5°C ± 4.5°C) and Lew/PerfadexPlus (30.5°C ± 3.1°C) after 15 min ESLP. Representative thermal lung imaging of the right lungs is shown in Supplementary Figures 1A, B. Before SCS began, mean temperature was 18.6°C, which decreased to 9.8°C during the 120 min cooling period. These values are exemplary, as there were no significant differences between the test groups (Figure 1C).

### Blood Gas Analyses Revealed Temperature-Dependent Metabolic Activities With Significant Differences Based on Perfusion Solution Compositions

**FIGURE 4:**
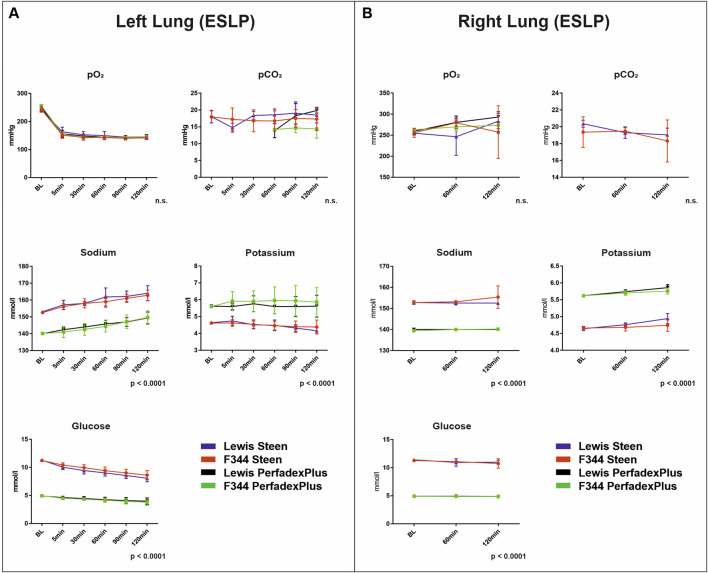
Blood gas analyses. Time-dependent representation of pO_2_ (mmHg), pCO_2_ (mmHg), sodium (mmol/L), potassium (mmol/L), and glucose (mmol/L) of left lungs under ESLP **(A)** and right lungs under SCS **(B)**. Data are expressed as mean ± SD. Statistical significance is indicated if *p* < 0.05 or as “n.s” if there is no significance.

Left lung blood gas analyses revealed no significant differences for pO_2_ (164.0 ± 2.5 mmHg) and pCO_2_ (16.7 ± 1.6 mmHg), with constant values over the entire ESLP (Figure 4A). Sodium levels increased over the course of ESLP in all groups, with STEEN solution groups starting at 152.8 mmol/L (Lew/STEEN 153.0 ± 0.7 mmol/L; F344/STEEN 152.6 ± 0.6 mmol/L) and increasing to 163.4 mmol/L (Lew/STEEN 164.0 ± 4.5 mmol/L; F344/STEEN 162.8 ± 3.1 mmol/L), while PerfadexPlus groups started at 140.1 mmol/L (Lew/PerfadexPlus 140.0 ± 0 mmol/L; F344/PerfadexPlus 140.2 ± 0.5 mmol/L) and increased to 149.5 mmol/L (Lew/PerfadexPlus 149.4 ± 3.5 mmol/L; F344/PerfadexPlus 149.6 ± 4.0 mmol/L), so that significant differences were observed between different perfusion solutions but not between different rat strains. The same significant differences were found for potassium, which had significantly higher levels with 5.8 mmol/L in PerfadexPlus groups (Lew/PerfadexPlus 5.6 ± 0.1 mmol/L; F344/PerfadexPlus 5.9 ± 0.1 mmol/L), compared to 4.5 mmol/L in STEEN groups (Lew/STEEN 4.5 ± 0.2 mmol/L; F344/STEEN 4.5 ± 0.1 mmol/L), with potassium levels remaining almost constant in all groups. In terms of glucose concentration, STEEN groups had higher values at 9.6 mmol/L (Lew/STEEN 9.4 ± 1.2 mmol/L; F344/STEEN 9.8 ± 0.9 mmol/L), which decreased to 8.4 mmol/L during ESLP (Lew/STEEN 8.1 ± 0.6 mmol/L; F344/STEEN 8.6 ± 0.8 mmol/L), while PerfedexPlus groups started at 4.9 mmol/L (Lew/PerfadexPlus 4.9 ± 0.1 mmol/L; F344/PerfadexPlus 5.0 ± 0.1 mmol/L) and dropped to 4.4 mmol/L (Lew/PerfadexPlus 4.9 ± 0.1 mmol/L; F344/PerfadexPlus 3.8 ± 0.6 mmol/L), also showing a significant difference between perfusion solutions but not between rat strains.

For right lungs, no significant differences between pO_2_ (Lew/STEEN 261.5 ± 19.3 mmHg; F344/STEEN 264.2 ± 13.4 mmHg; Lew/PerfadexPlus 277.6 ± 17.0 mmHg; F344/PerfadexPlus 268.8 ± 5.4 mmHg) and pCO_2_ (Lew/STEEN 19.6 ± 0.7 mmHg; F344/STEEN 19.1 ± 0.6 mmHg; Lew/PerfadexPlus 12.2 ± 0 mmHg; F344/PerfadexPlus 12.0 ± 0 mmHg) were seen (Figure 4B). Compared to left lungs, initial values of sodium (Lew/STEEN 152.8 ± 0.8 mmol/L to 152.6 ± 0.6 mmol/L; F344/STEEN 152.8 ± 0.5 mmol/L to 155.4 ± 5.4 mmol/L; Lew/PerfadexPlus 140.0 ± 0.7 mmol/L to 140.0 ± 0 mmol/L; F344/PerfadexPlus 139.4 ± 0.6 mmol/L to 140.2 ± 0.5 mmol/L), potassium (Lew/STEEN 5.6 ± 0.04 mmol/L to 5.9 ± 0.09 mmol/L; F344/STEEN 4.7 ± 0.05 mmol/L to 4.7 ± 0.2 mmol/L; Lew/PerfadexPlus 4.6 ± 0.05 mmol/L to 4.9 ± 0.15 mmol/L; F344/PerfadexPlus 5.6 ± 0.04 mmol/L to 5.8 ± 0.09 mmol/L), and glucose (Lew/STEEN 11.4 ± 0.12 mmol/L to 10.9 ± 0.4 mmol/L; F344/STEEN 11.3 ± 0.04 mmol/L to 10.8 ± 0.8 mmol/L; Lew/PerfadexPlus 4.9 ± 0.08 mmol/L to 4.9 ± 0.1 mmol/L; F344/PerfadexPlus 4.9 ± 0 mmol/L to 4.8 ± 0.05 mmol/L) remained constant during SCS. However, there were again significant differences between perfusion solutions but not rat strains.

Lactate was in the physiological range <0.5 mmol/L in all test groups, so that graphical representation was omitted. A different basic composition of STEEN and PerfadexPlus is shown in Supplementary Table S1.

### COP During ESLP Is Not for a Decisive Cause of Pulmonary Oedema Development

**FIGURE 5:**
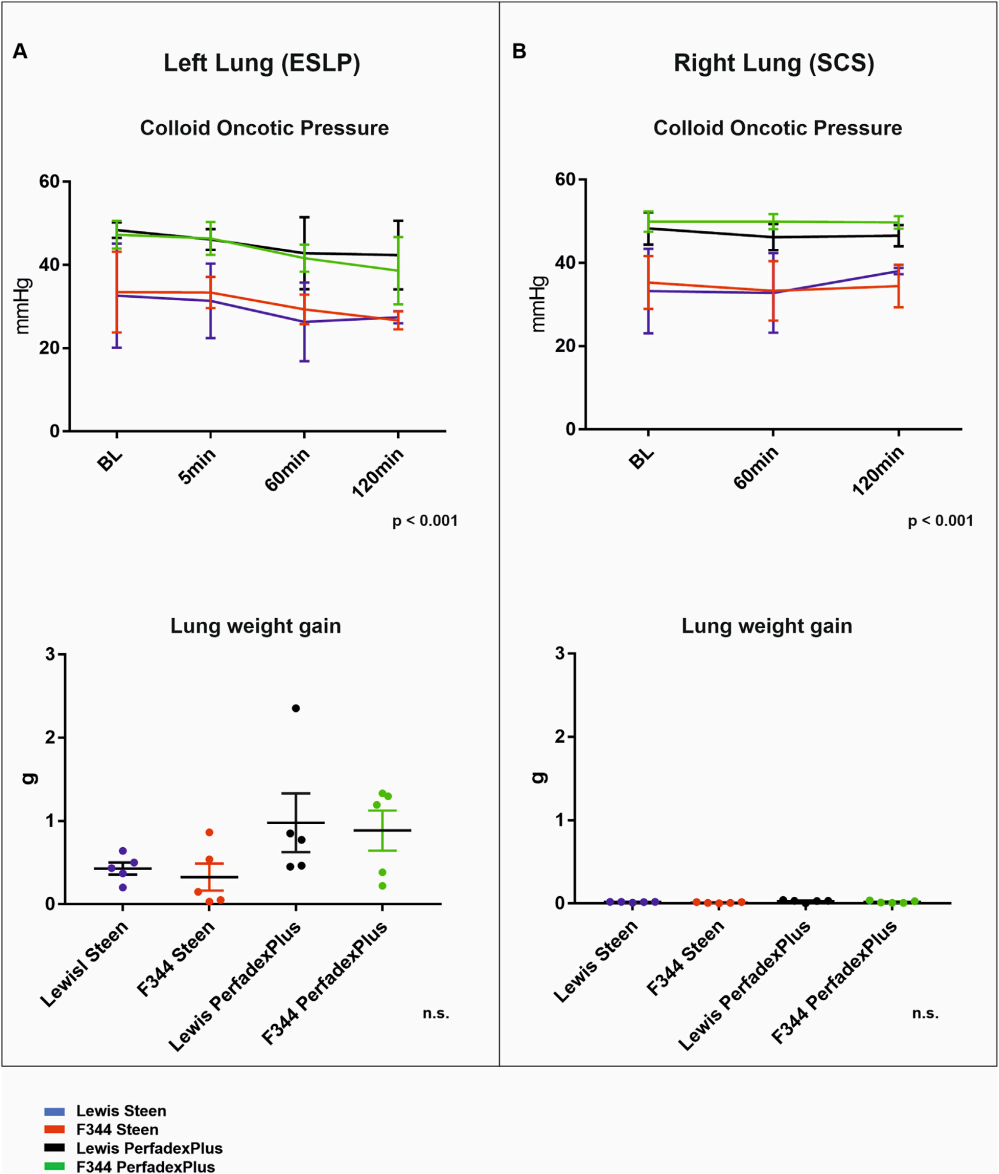
Colloid oncotic pressure and lung weight gain. Time-dependent representation of colloid oncotic pressures (COD) and lung weight gain (g) of left lungs under ESLP **(A)** and right lungs under SCS **(B)**. Data are expressed as mean ± SD for COD and mean ± SEM for lung weight gain. Statistical significance is indicated if *p* < 0.05 or as “n.s” if there is no significance.

COP also differed significantly between perfusion solutions but not between rat strains in ESLP lungs. Thus, initial and final COP were significantly lower in STEEN groups (Lew/STEEN 32.6 ± 12.5 mmHg and 27.4 ± 1.5 mmHg; F344/STEEN 33.5 ± 9.7 mmHg and 26.7 ± 2.1 mmHg) than in PerfadexPlus groups (Lew/PerfadexPlus 48.4 ± 1.9 mmHg and 42.4 ± 8.3 mmHg; F344/PerfadexPlus 47.3 ± 3.4 mmHg and 38.6 ± 8.1 mmHg). In comparison, weight gain in PerfadexPlus groups (Lew/PerfadexPlus 1.0 ± 0.4g and F344/PerfadexPlus 0.9 ± 0.5g) was considerably higher than in STEEN groups (Lew/STEEN 0.4 ± 0.07g and F344/STEEN 0.3 ± 0.16g) (Figure 5A). Comparable significant differences between STEEN and PerfadexPlus groups (Lew/STEEN 33.2 ± 5.1 mmHg and 38.0 ± 0.4 mmHg; F344/STEEN 35.3 ± 6.3 mmHg and 34.4 ± 2.5 mmHg; Lew/PerfadexPlus 48.3 ± 1.75 mmHg and 46.5 ± 1.2 mmHg; F344/PerfadexPlus 49.9 ± 1.1 mmHg and 49.7 ± 0.7 mmHg) were observed in COP analysis of SCS lungs, with no weight gain in either group (Figure 5B).

### Histological Examination Confirmed Optimal Constellation of Lung Procurement and ESLP Protocol Using In-House-Manufactured ESLP System

**FIGURE 6:**
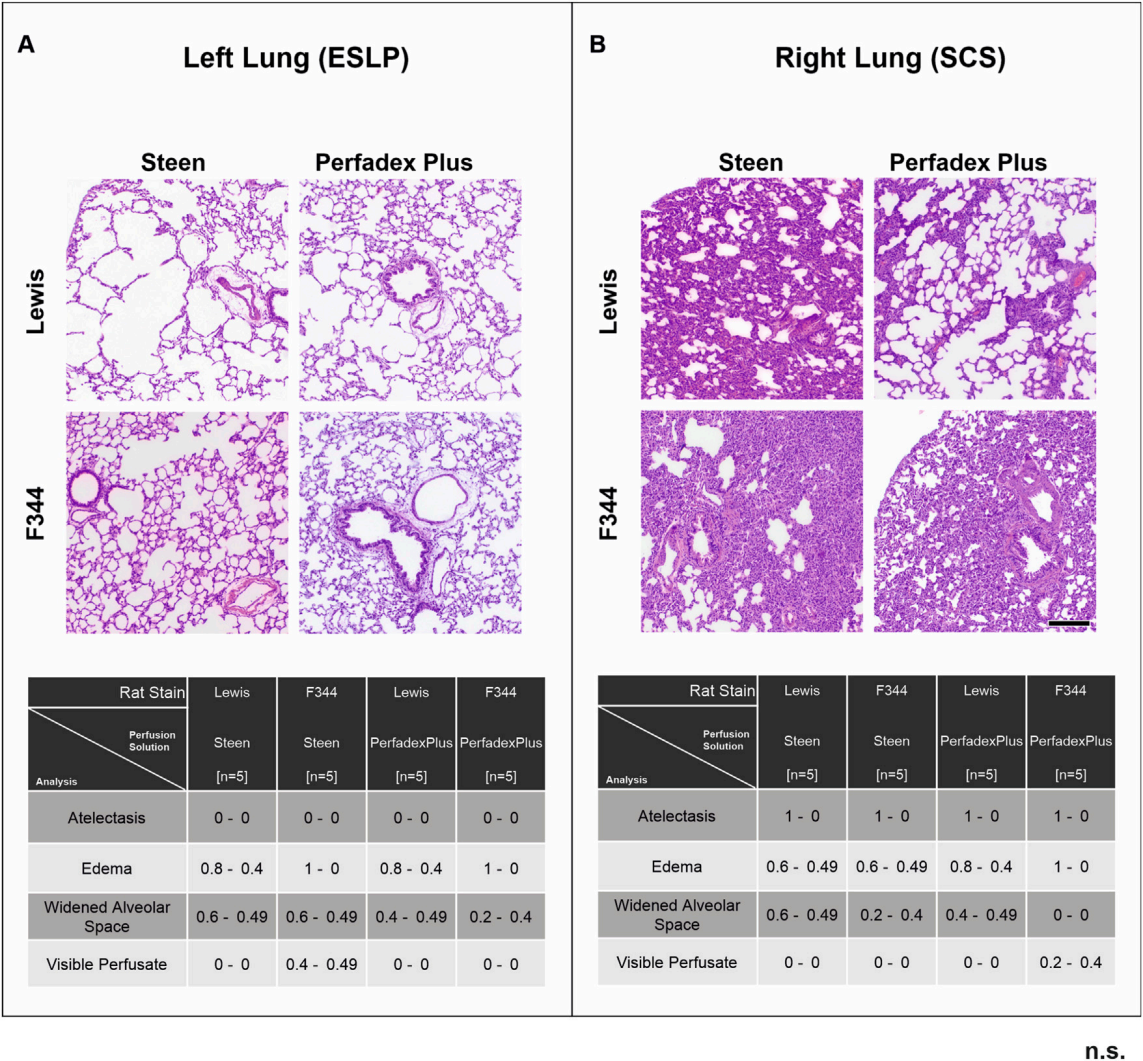
Overview of histological lung morphology and analysis of lung injury score. Representative images of hematoxylin-eosin-stained lungs (×50 magnification, scale bar 200 µm) and the classification of the lung injury score (0 = not present, 1 = present) by assessing the extent of atelectasis, oedema, widened alveolar spaces, and visible perfusate in the different test groups according to ESLP **(A)** and SCP **(B)**. Data are expressed as mean ± SD. Statistical significance is indicated if *p* < 0.05 or as “n.s” if there is no significance.

Histological evaluation showed essentially intact lung architecture with no signs of infarction or hemorrhage. There were different degrees of oedema (perivascular, intraalveolar, and interstitial), expansion of alveolar spaces, and intravascular perfusate, but these were not significant between the different perfusion groups and rat strains of ESLP and SCS. Only the frequency of atelectasis differed significantly: none of the perfused and ventilated ESLP lungs showed atelectasis, while all non-perfused, non-ventilated SCP lungs showed atelectasis (Figure 6).

### Cytokine and Chemokine Responses During 2-Hour ESLP

**FIGURE 7:**
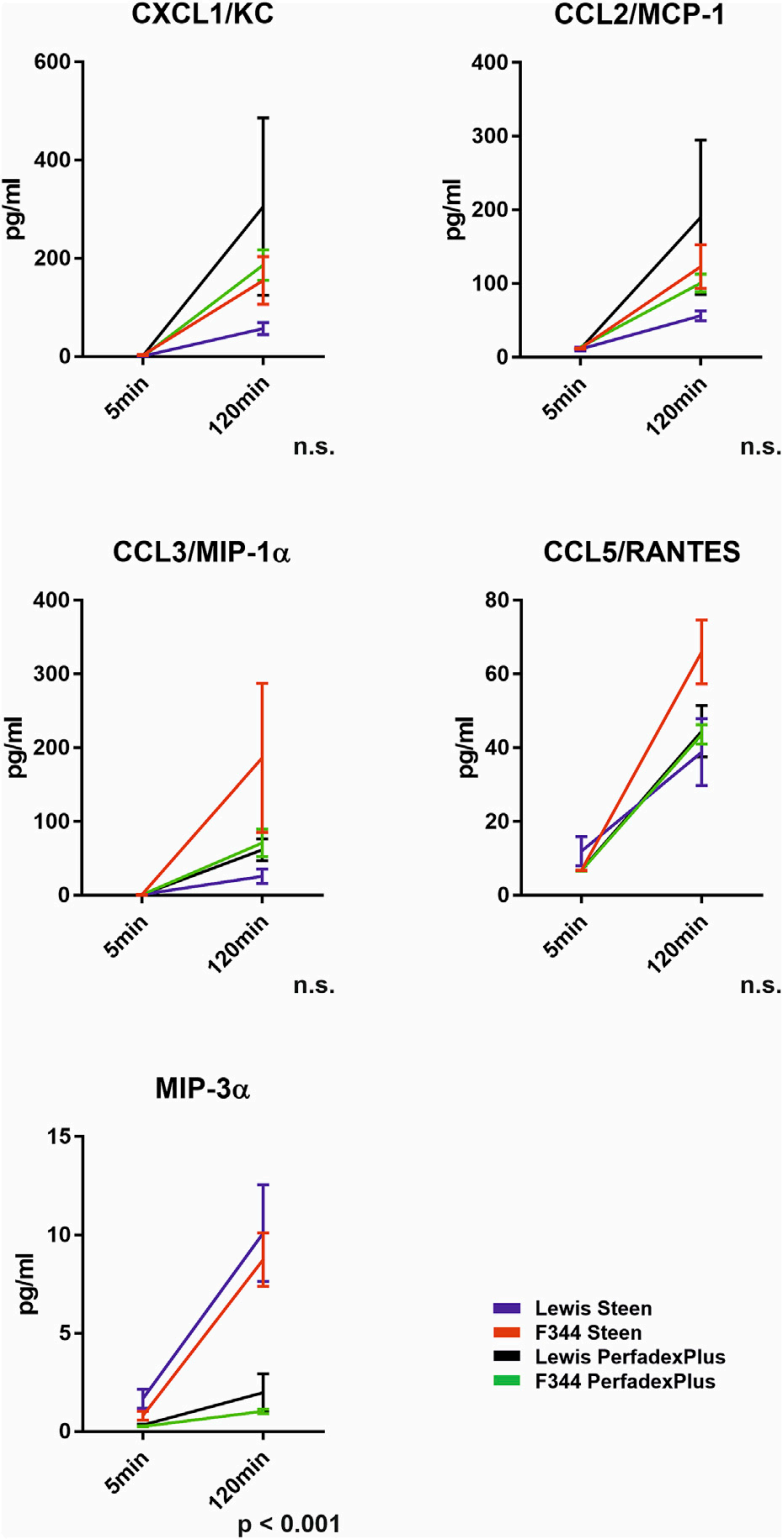
Cytokine and chemokine analysis in the ESLP perfusate. Time-dependent representation of CXCL1/KC (pg/mL), CCL2/MCP-1 (pg/mL), CCL3/MIP-1α (pg/mL), CCL5/RANTES (pg/mL), and MIP-3α (pg/mL) of the left lung under ESLP. Data are expressed as mean ± SEM. Statistical significance is indicated if *p* < 0.05 or as “n.s” if no significance.

A broad spectrum of cytokines and chemokines was measured during ESLP (Supplementary Table S2). However, a relevant increase during 2-hour ESLP was only observed for CXCL1/KC (Lew/STEEN 57.3 ± 12.4 pg/mL; F344/STEEN 155.3 ± 48.6pg/; Lew/PerfadexPlus 305.7 ± 180.4 pg/mL; F344/PerfadexPlus 186.7 ± 30.9 pg/mL), CCL2/MCP-1 (Lew/STEEN 56.3 ± 6.8pg/; F344/STEEN 123.1 ± 29.7 pg/mL; Lewis/PerfadexPlus 190.1 ± 104.8 pg/mL; F344/PerfadexPlus 100.9 ± 12.0 pg/mL), CCL3/MIP-1a (Lew/STEEN 25.8 ± 9.6 pg/mL; F344/STEEN 186.4 ± 101.1 pg/mL; Lew/PerfadexPlus 61.8 ± 14.7 pg/mL; F344/PerfadexPlus 71.0 ± 18.9pg/), CCL5/RANTES (Lew/STEEN 38.8 ± 9.1 pg/mL; F344/STEEN 66.0 ± 8.7 pg/mL; Lew/PerfadexPlus 44.5 ± 7.01 pg/mL; F344/PerfadexPlus 43.6 ± 2.7 pg/mL), and CCL20/MIP-3a (Lew/STEEN 10.1 ± 2.4 pg/mL; F344/STEEN 8.7 ± 1.3 pg/mL; Lew/PerfadexPlus 1.9 ± 0.9 pg/mL; F344/PerfadexPlus 1.0 ± 0.1 pg/m). The values of CCL20/MIP-3a differed significantly between perfusion groups (Figure 7). Anti-inflammatory cytokines like IL-10 were not detectable during ESLP or SCS, in which no pro-inflammatory markers were observed either.

## Discussion

Originally developed to significantly reduce IRI and improve the outcome after DLTx, normothermic ESLP is now increasingly used for organ reconditioning, repair, and reassessment prior to DLTx [3–8]. In this context, valid preclinical large and small animal models are required to conduct translationally relevant studies. Small animal models, in particular rat ESLP, offer a number of advantages, especially with regard to fundamental statements on new diagnostic and therapeutic ESLP strategies [12, 20]. For example, the comparatively inexpensive small animal experiments allow more detailed analysis of multiple variables in a larger sample size, leading to stronger treatment validation with greater certainty and less susceptibility to outlier error. This means that the scientific basis for subsequent porcine large animal experiments, which are better suited as translational models due to their phylogenetic proximity to humans, can be conducted in a much shorter time.

One of the most commonly used systems for this application is the commercially available ESLP system from Harvard Apparatus, which is designed for double-lung application by connecting the complete heart-lung block and whose initial priming volume of >150 mL is regularly exchanged in the open circuit at great expense, comparable to the Lund or Toronto protocol [3, 12, 21–23]. With the aim of having an ESLP available that is affordable both in purchase and for ongoing experiments, we have developed our self-designed ESLP that can be used for both double- and single-lung application, working with a priming volume of 17 mL in a closed circuit. In single-lung mode, it offers the possibility to obtain both ESLP lung and negative SCS control from one animal, from which a sufficient amount of perfusate/blood mixture for ESLP can be generated according to human protocols without the need to euthanize another donor animal, in contrast to the Harvard Apparatus for ESLP in small animals, which is described and used almost exclusively in the literature and, where >3 animals must be euthanized for >150 mL priming [14]. In addition, it can only be operated in double-lung mode, so that an additional animal is required to generate the SCS control, which is then not from the same animal and can lead to a certain bias in evaluation [12, 14, 16]. Overall, the use of our ESLP enables optimal compliance with the *reduction* of the 3Rs principles of animal research. For validation of our ESLP system and simultaneous establishment of a reliable, translation-relevant small animal model, we chose the rat strains Lew and F344, which are established for the induction of acute or chronic rejection reactions after transplantation [18, 19]. With PerfadexPlus and STEEN, we also used the gold standards and deliberately avoided further additives (e.g., Urbason) to provide relevant baseline data for future iterative experiments that will allow detailed and reliable differentiation between ESLP- and SCS-induced as well as exsitu perfusion- and transplantation-associated changes [8]. Since our system fulfils the technical requirements and the literature almost exclusively describes the Lund or Toronto protocol with upstream warm or cold ischemia prior to ESLP, we decided to imitate the OCS protocol, supplemented by important potential improvements (e.g., systolic PAP and thermography) and standards defined in the literature for ESLP application in small animals and the adaptation of relevant parameters for the single-lung application tested here [4–6, 17, 24, 25].

Part of our study was also to establish our low-volume ESLP with an associated basic protocol, whereby we deliberately avoided the addition of potentially protective drugs in order to be able to make an unbiased statement about which constellation of transplant-relevant rat strains with which perfusion solution represents the best basic protocol. In the literature, ESLP times of 1–6 h are reported for small animal models, but ESLP must be terminated within the first hour in the event of massive oedema formation if no protective drugs such as Urbason are administered, so that an ESLP duration of 120 min was considered sufficient in our context [23]. The ESLP key parameters remained constant overall, confirming the successful establishment of a valid, reproducible system that can be used reliably in both rat strains and regardless of the perfusate used. The target flow of 20% of cardiac output defined in the literature was halved for the single-lung application in order to avoid hyperperfusion with consecutive hydrostatic oedema formation [13, 22, 23]. In addition to the physiological PAP, the barely measurable lactate values also confirmed sufficient perfusion in our ESLP for adequate cell supply.

As a non-invasive method, thermography can generally be used in ESLP in a variety of ways to not only detect but also localize areas with poor perfusion via thermal changes in surface temperature both in the rewarming and preservation phase, thus predicting lung function and differentiating between transplantable and non-transplantable lungs [26, 27]. The implementation of this technique in our protocol once again confirmed sufficient perfusion of the single lung due to the homogeneous surface temperature shown. Another advantage, especially of time-dependent thermography, was that we started ventilation when physiological lung temperature was reached, preventing temperature-dependent barotrauma, which was confirmed by histologically intact lung architecture and would otherwise have accelerated oedema formation [26, 27]. This cannot be guaranteed with the usual ESLP procedures of starting ventilation at a perfusate temperature of 32°C (XPS) or 34°C (OCS), or even independently of this at a specific time, as the systemic temperature loss can only be effectively compensated through precise technical coordination, e.g., in our case of the double-walled organ chamber, the heat exchanger and the 42-degree water bath, so that physiological lung temperature was guaranteed over the entire ESLP [3–8, 22].

In addition to effective perfusion and organ temperature, sufficient oxygen supply is the missing key component of valid ESLP to maintain cell metabolism, which was ensured by our single-lung-adapted ventilation. Thus, energy production was achieved via aerobic glycolysis, which was reflected in the constant glucose consumption, continuous CO_2_-increase within the citrate cycle, and the physiological, barely measurable lactate values [12, 23].

In ESLP, hyperoncotic perfusion solutions are used as standard to prevent oedema formation through intravascular COP increase based on albumin (STEEN) or dextran (PerfadexPlus), with the latter having significantly higher COP [28]. However, since the PerfadexPlus groups showed a significantly higher weight gain during ESLP, and our data could exclude hyperperfusion, pathologically increased intravascular pressure, and microinjury-induced barotrauma as causes, both dextransensitivity described for rats and the time-, species-, and temperature-specific cytokine profile could be responsible for the weight gains we observed [16, 20, 22]. Analogous to the literature, we found a chemokine-heavy profile which, in addition to chemotaxis, could favorably influence oedema formation, in particular through the associated diapedesis [29]. Although weight gains of up to 30% described in the literature are comparable to our data, the omission of oedema-protective drugs (e.g., Urbason) led to the termination of the experiment within the first hour of ESLP in these studies due to massive oedema formation with fluid shift from intravascular to intraalveolar and interstitial and a resulting increase in COP [21–25]. In contrast, our study revealed a decrease in COP during the 2-h ESLP, confirming a stable and valid ESLP with associated standard protocol even without protective medication. As part of our ESLP-oriented protocol optimization, we prospectively opted for the systolic PAP as the perfusion-limiting factor (<30 mmHg), which is also within the physiological range of the mean pulmonary artery pressure (mPAP) <20 mmHg frequently used in the literature. However, the sPAP as the peak pressure acting in the vascular system has a greater influence on oedema formation, i.e., the fluid shift from intravascular to interstitial, than the mPAP, so that the positive influence of the sPAP could have come into play here and could be of greater importance for ESLP than the mPAP [30]. However, it should ultimately be noted that ESLP-related oedema is absorbed to a certain extent post-transplant and is therefore irrelevant for PGD score and outcome after DLTx [11, 21].

## Data Availability

The raw data supporting the conclusions of this article will be made available by the authors, without undue reservation.

## Supplementary Material

Supplementary Figure S1 Thermographical data acquisition and analysis of SCS lungs Representative native photos and corresponding thermal images of the lungs at the different time points as well as the respective average temperature are shown **(A,B)**. Graphical representation shows the surface temperatures of the different test groups over time **(C)**. Data are expressed as mean ± SD. Statistical significance is indicated if *p* < 0.05, or as “n.s” if there is no significance.

Supplementary Figure S2 Detailed histological findings of the lung injury score. Analyzed lung samples showed the following changes to varying degrees: Perivascular oedema **(A)**, especially around the pulmonary arteries (arrowheads); intra-alveolar oedema [**(B)**, arrowhead] and thickened alveolar septa as signs of interstitial oedema [**(C)**, arrowhead]; further widening of the alveolar spaces, as a sign of over ventilation [**(D)**, arrowhead]; atelectasis **(E)**; and, in rare cases, the presence of perfusion solution [**(F)**, arrowhead] were noted in HE staining. Magnification: **(A, D)** = ×50, **(B, C, E)** = ×100, and **(F)** = ×200. Scale bars: **(A, D)** 200 µm, **(B, C, E)** 100 µm, and **(F)** 20 µm.

## Abbreviations

BGA, Blood gas analysis; BW, Body weight; COP, Colloid oncotic pressure; DLTx, Double lung transplantation; ESLP, *Ex situ* lung perfusion; F344, Fischer F344 rat; HE, Hematoxylin and eosin staining; I:E, Inspiration:expiration time; IRI, Ischemia/reperfusion injury; LA, Left atrium; Lew, Lewis rat; Min, Minutes; OCS, Organ Care System Lung; PA, Pulmonary artery; PAP, Pulmonary artery pressure; PEEP, Positive end-expiratory pressure; RR, Respiratory rate; SCS, Static cold storage; THAM, Tris(hydroxymethyl)aminomethane; TV, Tidal volume.
