# Supplementary material for: Low-Volume Ex Situ Lung Perfusion System for Single Lung Application in a Small Animal Model Enables Optimal Compliance With “Reduction” in 3R Principles of Animal Research
Source: Transpl Int. 2024 Sep 9;37:13189. doi: 10.3389/ti.2024.13189 (PMC11418019; doi:10.3389/ti.2024.13189)
Supplement: Supplementary file 1 [file Table1.DOCX]

| **Perfusion Solution**    **Parameter** | **STEEN** | **Perfadex Plus** |
| --- | --- | --- |
| **pH** | 7.483 | 6.638 |
| **Potassium [mmol±L]** | 4.6 | 5.6 |
| **Sodium [mmol±L]** | 152 | 141 |
| **Glucose [mmol±L]** | 10.7 | 4.8 |
| **Colloid Oncotic Pressure [mmHg]** | 38 | 45.7 |

Tab 1. Perfusion Solution Baseline

Tab 2. Cytokines in perfusate after 2h ESLP

| **Strain  Analyte** | **Lew/STEEN** | | **F344/STEEN** | | **Lew/PerfadexPlus** | | **F344/PerfadexPlus** | |
| --- | --- | --- | --- | --- | --- | --- | --- | --- |
|  | **5 Min** | **120 Min** | **5 Min** | **120 Min** | **5 Min** | **120 Min** | **5 Min** | **120 Min** |
|  | **Mean ± SEM** | **Mean ± SEM** | **Mean ± SEM** | **Mean ± SEM** | **Mean ± SEM** | **Mean ± SEM** | **Mean ± SEM** | **Mean ± SEM** |
| **G-CSF** | 0.9 ± 0.5 | 0.39 ± 0.04 | 0.4 ± 0.0 | 0.4 ± 0.0 | 0.42c± 0.1 | 0.4 ± 0.3 | 0.5 ± 0.0 | 0.5 ± 0.1 |
| **GM-CSF** | 90.8 ± 90.0 | 0.87 ± 0.0 | 0.9 ± 0.0 | 0.9 ± 0.0 | 0.9 ± 0.0 | 2.5 ± 1.0 | 1.1 ± 0.2 | 1.3 ± 0.4 |
| **IFN-γ** | 10.3 ± 0.0 | 10.3 ± 0.0 | 10.3 ± 0.0 | 10.3 ± 0.0 | 10.3 ± 0.0 | 10.3 ± 0.0 | 24.5 ± 14.2 | 10.3 ± 0.0 |
| **IL1-α** | 18.0 ± 15.9 | 2.1 ± 0.2 | 2.4 ± 0.3 | 2.5 ± 0.2 | 2.1 ± 0.4 | 2.2 ± 0.3 | 2.0 ± 0.2 | 3.7 ± 0.7 |
| **IL1-β** | 130.0 ± 128.5 | 1.8 ± 0.4 | 2.1 ± 0.2 | 1.7 ± 0.2 | 2.2 ± 0.3 | 4.7 ± 1.2 | 1.2 ± 0.2 | 2.3 ± 0.5 |
| **IL-2** | 213.6 ± 148.5 | 72.3 ± 4.3 | 65.2 ± 0.0 | 65.2 ± 0.0 | 65.2 ± 0.0 | 165.7 ± 68.0 | 65.2 ± 0.0 | 116.0 ± 45.8 |
| **IL-4** | 15.0 ± 14.2 | 1.7 ± 0.6 | 1.2 ± 0.2 | 1.1 ± 0.3 | 1.9 ± 0.5 | 1.1 ± 0.3 | 0.8 ± 0.0 | 1.4 ± 0.2 |
| **IL-5** | 51.4 ± 41.0 | 8.8 ± 0.4 | 10.9 ± 1.8 | 9.7 ± 0.4 | 8.4 ± 0.9 | 12.0 ± 1.7 | 10.9 ± 1.7 | 16.0 ± 3.6 |
| **IL-6** | 104.0 ± 40.0 | 64.0 ± 0.0 | 64.0 ± 0.0 | 64.0 ± 0.0 | 64.0 ± 0.0 | 94.7 ± 30.8 | 64.0 ± 0.0 | 64.0 ± 0.0 |
| **IL-7** | 104.0 ± 103.0 | 0.4 ± 0.0 | 0.6 ± 0.2 | 0.5 ± 0.1 | 0.7 ± 0.2 | 5.1 ± 2.2 | 0.9 ± 0.3 | 4.1 ± 0.9 |
| **IL-10** | 17.6 ± 10.5 | 7.1 ± 0.0 | 7.1 ± 0.0 | 7.1 ± 0.0 | 7.1 ± 0.0 | 7.1 ± 0.0 | 7.1 ± 0.0 | 7.1 ± 0.0 |
| **IL-12** | 56.0 ± 53.6 | 3.0 ± .06 | 2.4 ± 0.0 | 2.4 ± 0.0 | 3.2 ± 0.8 | 4.6 ± 1.5 | 2.4 ± 0.0 | 2.4 ± 0.0 |
| **IL-13** | 32.6 ± 25.0 | 7.7 ± 0.0 | 7.7 ± 0.0 | 7.7 ± 0.0 | 7.7 ± 0.0 | 10.9 ± 3.3 | 7.7 ± 0.0 | 7.7 ± 0.0 |
| **IL-17A** | 7.7 ± 5.0 | 2.7 ± 0.0 | 2.7 ± 0.0 | 2.7 ± 0.0 | 2.9 ± 0.3 | 2.5 ± 0.2 | 2.7 ± 0.0 | 3.0 ± 0.3 |
| **IL-18** | 1343.0 ± 651.0 | 657.3 ± 119.6 | 757.4 ± 63.5 | 384.3 ± 71.8 | 877.7 ± 90.4 | 999 ± 97.0 | 699.6 ± 52.0 | 874.7 ± 68.4 |
| **M-CSF** | 1.9 ± 0.0 | 1.5 ± 0.2 | 1.6 ± 0.2 | 1.7 ± 0.2 | 1.8 ± 0.2 | 1.8 ± 0.0 | 1.7 ± 0.2 | 1.4 ± 0.2 |
| **TNF-α** | 68.2 ± 42.3 | 31.0 ± 3.2 | 31.4 ± 1.8 | 81.6 ± 38.3 | 33.3 ± 3.6 | 79.2 ± 28.2 | 29.7 ± 0.0 | 59.5 ± 14.0 |
| **VEGF** | 13.3 ± 12.8 | 0.5 ± 0.0 | 0.5 ± 0.0 | 0.5 ± 0.0 | 0.5 ± 0.0 | 14.3 ± 11.5 | 0.5 ± 0.0 | 1.9 ± 0.9 |

n.s.
