# Supplementary figures and images for: Low-Volume Ex Situ Lung Perfusion System for Single Lung Application in a Small Animal Model Enables Optimal Compliance With “Reduction” in 3R Principles of Animal Research
Source: Transpl Int. 2024 Sep 9;37:13189. doi: 10.3389/ti.2024.13189 (PMC11418019; doi:10.3389/ti.2024.13189)

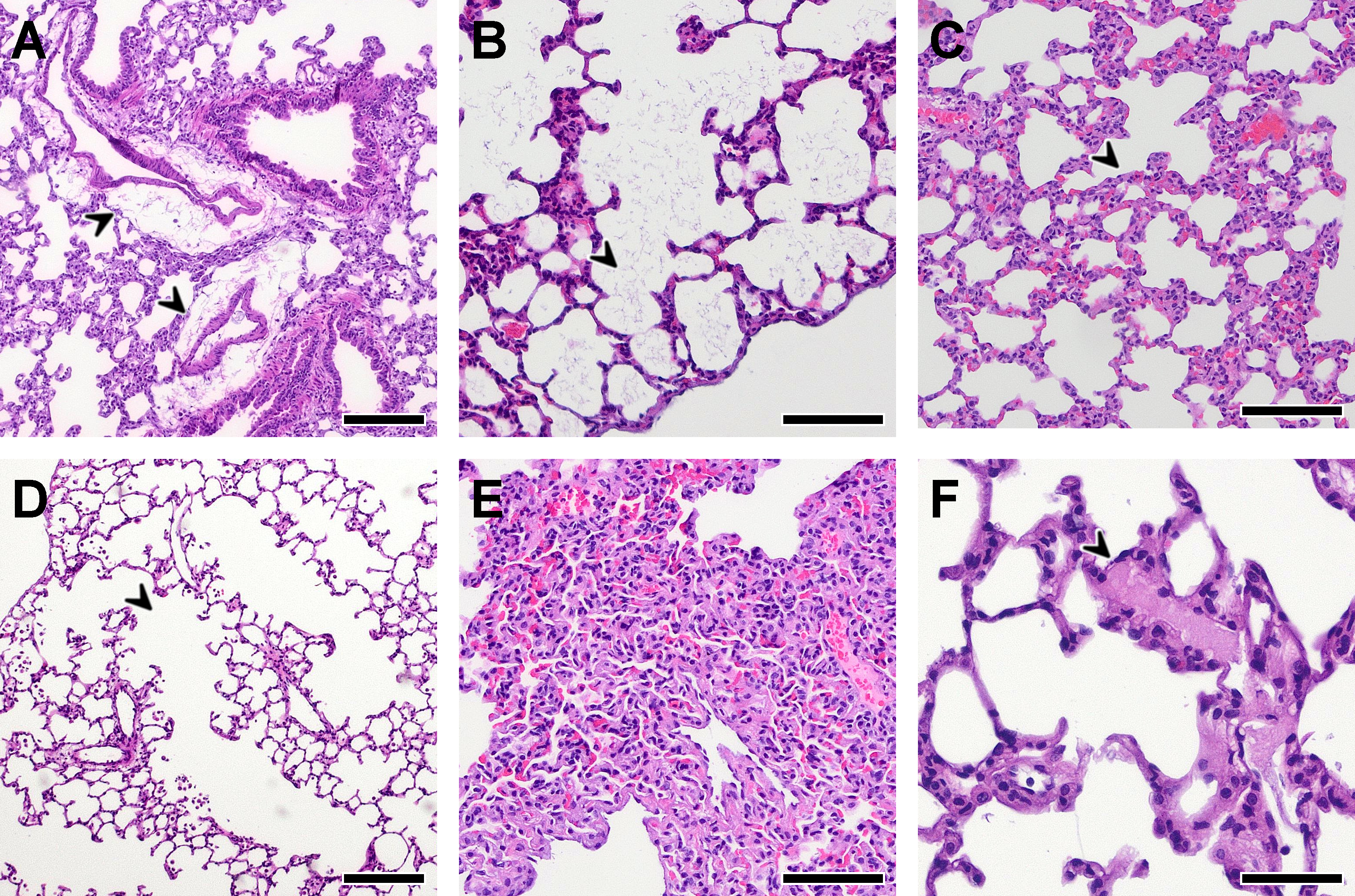

Supplement: Supplementary file 2 [file Image1.TIF]

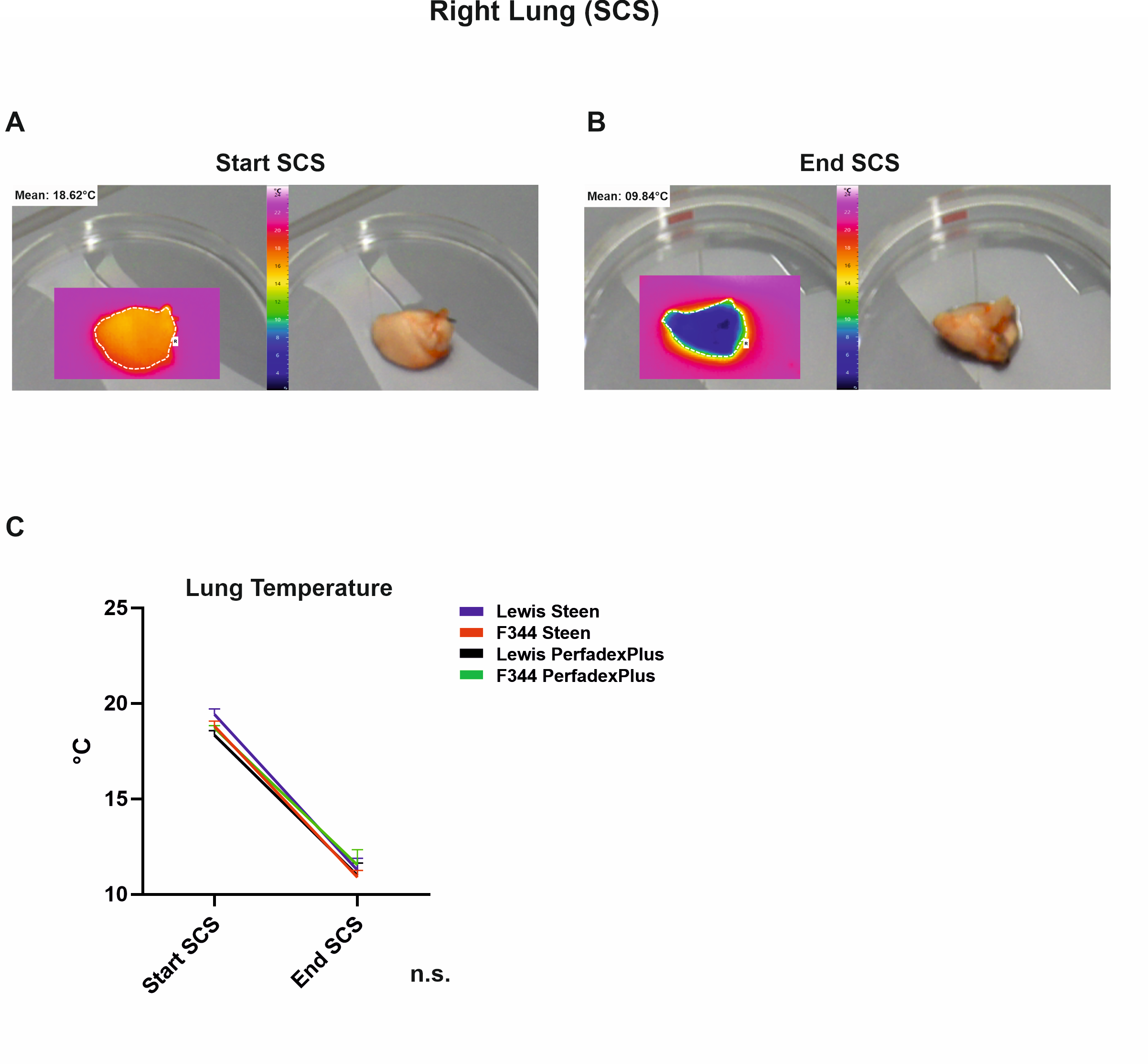

Supplement: Supplementary file 3 [file Image2.TIFF]
